# Clinical response to systemic AAV gene therapy in a large animal model of late-stage lysosomal storage disease

**DOI:** 10.1038/s41434-026-00618-0

**Published:** 2026-05-04

**Authors:** Jacqueline E. Hunter, Caitlyn M. Molony, Dana L. Clarke, Wojciech Panek, Charles H. Vite, Sanjeev Chawla, Harish Poptani, John H. Wolfe

**Affiliations:** 1 Research Institute of Children’s Hospital of Philadelphia, Philadelphia, PA USA; 2 W.F. Goodman Center for Comparative Medical Genetics, School of Veterinary Medicine, University of Pennsylvania, Philadelphia, PA USA; 3 Department of Radiology, Perelman School of Medicine, University of Pennsylvania, Philadelphia, PA USA; 4 Department of Molecular and Clinical Cancer Medicine, University of Liverpool, Liverpool, UK; 5 Department of Pediatrics, Perelman School of Medicine, University of Pennsylvania, Philadelphia, PA USA; 6 Present Address: Department of Small Animal Clinical Sciences, College of Veterinary Medicine, University of Florida, Gainesville, FL USA

**Keywords:** Metabolic disorders, Neurological disorders

## Abstract

The benefit of early diagnosis and treatment has been demonstrated in animal models of several lysosomal storage diseases. In a clinical setting, however, diagnoses are often not made until after patients become symptomatic. The lysosomal storage disease alpha-mannosidosis is caused by a genetic deficiency of lysosomal alpha-mannosidase, leading to the widespread presence of storage lesions throughout the brain and other tissues. In a feline model of alpha-mannosidosis, we previously demonstrated complete correction of the brain following delivery of AAVhu.32-fMANB via the carotid artery in the early symptomatic stage. Here, we investigate the efficacy of AAV gene therapy on globally distributed storage lesions in animals with advanced disease. Some improvements in clinical parameters were observed, however these improvements were less than in animals with less advanced disease. Although the treated animals were improved compared to untreated animals, increasing the vector dose did not further improve clinical outcomes. These results further demonstrate the importance of early detection and treatment of a lysosomal storage disease to successful outcomes. Despite this, partial correction extended the lifespan of diseased cats and may be medically beneficial to patients by slowing or stabilizing the progressive degenerative course of disease.

## Introduction

The lysosomal storage disease alpha-mannosidosis (AMD) occurs when lysosomal α-mannosidase (MANB) is either absent or defective due to mutations in the *MAN2B1* gene. Clinical features of AMD include central nervous system (CNS) involvement with intellectual disability, hearing impairment, coarse facial features, skeletal abnormalities, hepatosplenomegaly, and immunodeficiency [1, 2]. Mild, moderate, and severe clinical subtypes of AMD have been described [3, 4]. The most severe cases are typically diagnosed in infants, whereas milder forms of the disease may be diagnosed later in childhood or as late as adulthood. AMD-causing mutations can occur anywhere in the *MAN2B1* gene, with no correlation between genotype and clinical phenotype [3].

Several naturally occurring AMD animal models have been identified, which have proven invaluable in evaluating potential new therapies for AMD. To date, AMD has been documented in cattle, cats, and guinea pigs [5–8]. In addition to these animal models, a mouse *MAN2B1* knockout has also been generated [9], however, even though the mouse model lacks MANB activity, it has sparse storage lesions and does not exhibit an overt clinical phenotype. The feline AMD model has a severe phenotype and is comparable to severe AMD in humans [2, 5, 10].

Currently available therapies for AMD include hematopoietic stem cell transplantation (HSCT) and enzyme replacement therapy (ERT) [11]. ERT is an effective AMD treatment with fewer risks than HSCT [12–14], however it requires weekly administration, and the enzyme is not able to cross the blood-brain barrier (BBB). Thus, it is only approved for the treatment of mild-to-moderate cases of AMD that do not have CNS involvement. For treating AMD with CNS involvement, AAV-based therapeutics have been successful in correcting storage in the brain in preclinical studies [15–18].

Better clinical outcomes have been demonstrated following early treatment in several lysosomal storage disease models. Presymptomatic treatment with AAV resulted in an improved therapeutic response when compared to treatment at symptomatic time points in  animal models of Fabry disease [19], metachromatic leukodystrophy [20], late infantile neuronal ceroid lipofuscinosis [21, 22], and MPS VII [23–25]. While animal models allow for treatment at pre-symptomatic time points, diagnosis is often delayed in human patients. The initial signs and symptoms, such as hearing loss and recurrent infections, are not AMD-specific, which can lead to a delay in diagnosis [1, 4]. One study comparing outcomes in infants with Krabbe disease that received umbilical cord blood transplantation at either pre- or post-symptomatic time points demonstrated better outcomes for infants treated pre-symptomatically [26]. Although long-term follow up showed improvement in some symptoms including cognition, most were out of the normal range [27]. Another study in siblings with AMD showed similar benefits with HSCT [28].

The current study assessed the effect of vector dose on clinical outcomes in a large animal model of advanced AMD. AMD cats become symptomatic at approximately 5 weeks of age [16]. Previous studies have demonstrated significant clinical improvement when treating via the cisterna magna in the early symptomatic period at 6 weeks [15, 17]. However, treatment at two progressively more severe symptomatic time points (9 and 12 weeks) was less beneficial [29]. This study was limited by the vector dose that could be administered via the CM. To investigate whether response to treatment could be improved in symptomatic animals, the current study started with a dose that previously generated global correction of the cat brain with treatment via the carotid artery during the early symptomatic period [18]. Vector dose was increased by either 50% or 100% and the effect on clinical parameters was assessed.

## Methods and materials

### Plasmid and AAV production

Gene transfer cis-plasmids containing feline alpha-mannosidase (fMANB) gene were expressed from the minimal human GUSB promoter in the pZac vector that included an SV40 splice donor/acceptor signal and the bovine growth hormone polyadenylation signal, as described [30–33]. Recombinant AAVhu.32 vectors were packaged by the University of Pennsylvania Vector Core by triple transfection of HEK293 cells with the AAV cis-plasmid, AAV trans-plasmid containing AAV rep and cap genes, and adenovirus helper plasmid. Vectors were purified using iodixanol gradient ultracentrifugation, and the titers were determined by real time PCR [34].

### Animals and vector injections

All animal care and procedures were in accordance with the Institutional Animal Care and Use Committee at the University of Pennsylvania. Alpha-mannosidosis affected cats were produced in the breeding colony of the University of Pennsylvania School of Veterinary Medicine by carrier-to-carrier breeding. Intracarotid injections of cats were performed under anesthesia using a cut-down procedure to access the common carotid artery. Surgeries were performed at 12 weeks of age. The injected doses in cats were 5.0 × 1013 vg/kg, 7.5 × 1013 vg/kg, or 1.0 × 1014 vg/kg. Heart and respiratory rates were monitored until full recovery. All animals were injected by the same staff and using the same procedure. The surgeon and vivarium staff were all blind to the dose received. Animals were assessed daily by veterinarians or trained veterinary staff and were euthanized when they reached the humane endpoint. Criteria for euthanasia included (1) loss of 15% of body weight, (2) loss of responsiveness to human interaction, and (3) significant changes in behavior or attitude relative to what is normal monthly from treatment until euthanasia by the same blind observer who was unaware of whether cats were treated or not. Observers of clinical signs were blinded to the vector dose; but could not be blinded to the disease state of the animals due to the obvious physiognomic abnormalities.

### MANB enzymatic activity assay

MANB enzymatic activity was measured in serum, CSF or brain tissue as described [15–18, 35]. Tissues were homogenized in saline containing 0.2% Triton X-100, and MANB activity was determined using the substrate 4-methylumbelliferyl α-D-mannopyranoside in 0.1 M sodium citrate buffer (pH 4.4) at 37 °C for 1 h. Protein was quantified using bicinchoninic acid (BCA) assay.

### Tissue collection and preparation

Treated cats were allowed to live as long as possible and were euthanized when humane endpoint criteria were reached according to the IACUC protocol. Euthanasia was performed using an overdose of intravenous barbiturates and transcardially perfused with 0.9% cold saline. Tissues were drop-fixed in 4% paraformaldehyde for 48 hours. Euthanasia allowed these brains to be perfused with PBS and immediately transferred to paraformaldehyde in order to preserve brain morphology.

For cryosectioning, brains were cryoprotected in 30% sucrose, embedded in optimum cutting temperature solution (Sakura, Torrance, CA) and cryosectioned at 40 µm (Leica Microsystems, Wetzlar, Germany). For histology, tissues were paraffin embedded and stained with H&E or Luxol fast blue.

For serum collection, whole blood was allowed to clot for 30 min at room temperature and then centrifuged at 1000 x *g* for 15 min. The supernatant was then aspirated and stored at –80 °C. CSF was collected using a 22-gauge spinal needle from the cerebello-medullary cistern and stored at –80 °C.

### Real time PCR

Quantitative real time PCR was used to determine the viral genome copies present in the brain. Genomic DNA was extracted from 2 to 3 sections of each transverse brain block and the vector genome copies were quantified separately at each transverse level. Copies of fMANB vector genome were quantified using PowerUp SYBR Green Master Mix (Thermo Fisher, Austin, TX). Triplicate samples derived from each DNA pool were used for quantification. The primer sequences were as follows: fMANB forward: 5’-GGG AGG AGC ATG GCT AGA CA-3’, fMANB reverse: 5’-AAC AAC AGA TGG CTG GCA ACT-3’.

### MRI

All cats underwent MRI on a 3 T Tim Trio whole body MR scanner (Siemens, Erlangen, Germany) equipped with a single-channel 11-cm internal diameter transmit-receive birdcage coil (M2M). The anatomical imaging protocol included a 3-plane scout localizer to determine orientation and position of the brain. Additionally, axial T2-weighted images (repetition time /echo time = 2500/60 ms; section thickness = 2 mm; number of slices = 20; number of excitations = 1; and axial T1-weighted images (repetition time/echo time = 650/27 ms) were acquired. Physiological monitoring, including pulse oximetry and vital signs (oxygen saturation and heart rate), was recorded before and during the entire scanning period.

### Proton MRS imaging

Single slice 2D multivoxel proton MRS imaging (^1^H-MRS) was performed using a spin echo (point-resolved spectroscopy) sequence with water suppression using a chemical shift selective saturation (CHESS) sequence. Sequence parameters included repetition time/echo time (2500/30 ms), number of excitations (16), field of view (55  × 55 mm^2^), slice thickness (7 mm), bandwidth (1500 Hz), and matrix size (16 × 16). The typical voxel size was 3.4 × 3.4 × 7 mm^3^. The volume of interest was selected to include the different cortical and thalamic regions, avoiding the scalp. Four outer volume saturation slabs (30 mm thick) were placed outside the volume of interest to suppress lipid signals from the scalp. The dataset was acquired using elliptical k-space sampling with weighted phase encoding to reduce the acquisition time. Acquisition time for ^1^H-MRS sequence was 6:22 min. Manual shimming was performed to achieve an optimal value of full width half-maximum of the water signal. In general, a shimming of 520 Hz was achieved on the magnitude signal of the water resonance. Water unsuppressed ^1^H-MRS spectra were also acquired to use the water signal for computing relative metabolite ratios.

### DTI

DTI data were acquired using 30 non-collinear/non-coplanar directions with a single-shot spin-echo, echo-planar readout sequence. The sequence parameters were as follows: repetition time/echo time = 3000/75 ms, number of excitations = 8, field of view = 80 × 80 mm^2^, matrix size = 64 × 64, in-plane resolution = 1.25 × 1.25 mm^2^; slice thickness = 2 mm; b = 0, 1000 s/mm^2^, number of slices = 20, covering the whole brain.

### Data analysis

The ^1^H-MRS data were processed offline on a Leonardo workstation using the Syngo software (Siemens). For each spectrum, the acquired ^1^H-MRS signal (free induction decay) was zero-filled (2048 data points), smoothed (Hanning filter, width 200 ms), and Fourier-transformed. This was followed by phase (zero- and first-order polynomial) and baseline correction for optimal linear frequency dependence. Peak areas of oligosaccharide (3.4–3.9 ppm) and unsuppressed water (4.8 ppm) resonances were determined from each voxel encompassing the bilateral cortical and thalami regions. The fitting of the individual peak can be optimized by adjusting the chemical shift, amplitude, and line width interactively. Oligosaccharide peak areas were normalized with respect to unsuppressed water signal for each voxel. The metabolite ratios for oligosaccharide/water from 5 to 10 voxels that encompassed cortex and 4–6 voxels that encompassed thalamus were computed. The mean and SD values of metabolite ratios of oligosaccharide/water were reported from each region.

DTI data were processed and analyzed using DTI studio software (Johns Hopkins School of Medicine, www.mristudio.org). Pixel-wise fractional anisotropy (FA) maps were computed. FA color maps were used to draw the regions of interest from white matter regions (corpus callosum, external and internal capsule). These regions of interest were translated on FA maps to compute FA values, since FA was the only in vivo DTI parameter at 3 T that statistically distinguished normal from untreated AMD brains [36].

### Statistical analysis

Unpaired two-tailed Student’s *t* test and One-Way ANOVA with Bonferroni post-test were used, where applicable, to determine mean differences between groups. Data are reported as means ± SEM unless otherwise stated. For the ^1^H-MRS studies, the metabolite ratios of OS/water from cortical and thalamic regions were compared across different groups of animals (normal, untreated AMD, treated AMD) by one-way ANOVA with a post hoc Bonferroni test. Similarly, FA values as obtained from DTI data from corpus callosum, external capsule and internal capsule were compared across different groups of animals. All data analyses were performed using a statistical tool (SPSS for Windows, version 18.0; SPSS Inc., Chicago, III, USA).

## Results

Previously, it was demonstrated that delivery of AAVhu.32-fMANB to the carotid artery of 6-week-old cats affected by α-mannosidosis (AMD) produced complete correction of the brain at a high dose [18]. To evaluate the effect of advanced disease on efficacy of AAVhu.32-fMANB treatment, AMD affected cats were treated at 12 weeks of age, a time point that corresponds to intermediate stage disease. Animals received the same dose of vector (5.0 × 1013 vg/kg, *n* = 3) as used previously in 6-week-old cats [18]. Additional cohorts were treated with higher vector doses (7.5 × 1013 vg/kg or 1.0 × 1014 kg/vg, *n* = 3 for each group).

**Fig. 1:**
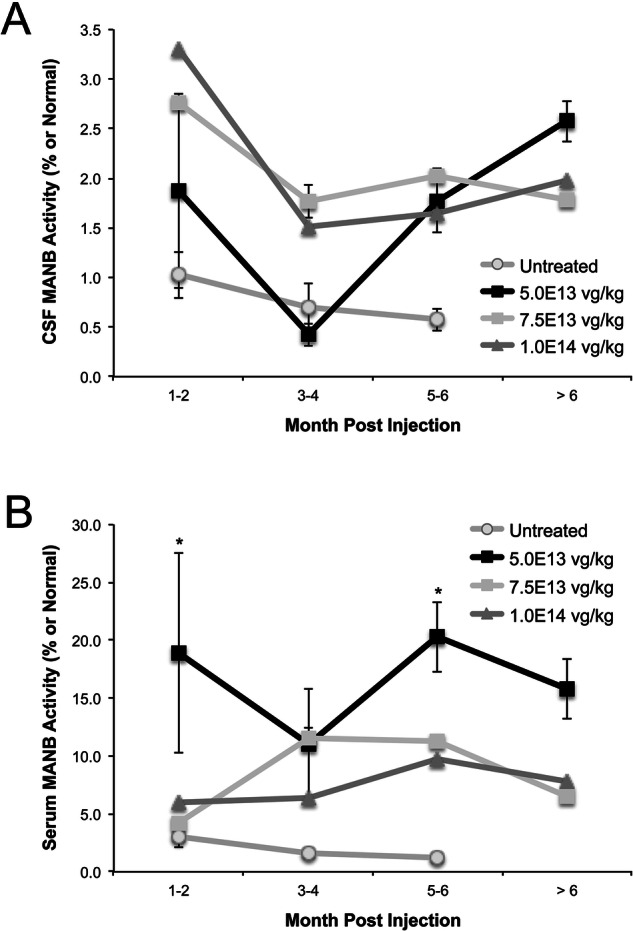
Effect of vector dose on MANB activity in CSF and serum of symptomatic AMD cats. CSF and serum samples were collected at regular intervals and assayed for MANB activity in both treated and untreated AMD cats. **A** CSF MANB activity was elevated compared to untreated cats up to 6 months post injection; however, none of the treatment groups animals had a significant increase. **B** Serum MANB activity was increased in treated cats compared with untreated animals, however, the increase was only significant in animals treated with 5.0 × 10^13^ vg/kg vector at 1–2 and 5–6 months post injection. There was no significant difference between other age groups and untreated animals. Note that comparisons to untreated cats cannot be made at time points greater than 6 months post injection as untreated cats do not live that long. Points represent mean + SEM. *p < 0.05.

At regular intervals following treatment, CSF and serum samples were collected to monitor levels of MANB activity in the treated cats. While CSF levels of MANB in all three treatment groups were elevated above that of untreated AMD cats, the increase did not reach significance at any time point for any dose group (Fig. 1A; significance at >6 months of age could not be determined as untreated animals do not live past 6 months of age). Serum levels of MANB remained elevated above that of untreated AMD cats for all treatment groups (Fig. 1B). Significance was reached for the 5.0 × 1013 vg/kg group at 1–2 months post injection and 5-6 months post injection (*P* < 0.01 for both time points). Of note, the CSF MANB levels for all three groups were lower than what was measured for animals treated with high dose (5.0  × 1013 vg/kg) vector at 6 weeks, whereas the serum MANB levels in the group treated at 12 weeks with the lowest vector dose (5.0 × 1013 vg/kg) was higher than what was measured in cats treated with high dose at 6 weeks [18].

Since the CSF and serum MANB levels were elevated over untreated animals for all treatment groups, we investigated the clinical effects. Animals were evaluated monthly for appearance of neurological symptoms including whole-body tremors and truncal ataxia. In untreated cats, these symptoms begin to manifest at approximately 5 weeks of age and become progressively worse until euthanasia is required due to their deteriorating condition. Whole body tremors and truncal ataxia remained mild in AMD cats treated at 6 weeks of age [18], whereas animals treated with the same vector dose (per kg body weight) at 12 weeks of age developed moderate to severe tremors and ataxia. Treatment at 12 weeks with higher vector doses did not prevent the development of tremors or ataxia, however both remained mild to moderate in all 3 dose groups.

**Fig. 2:**
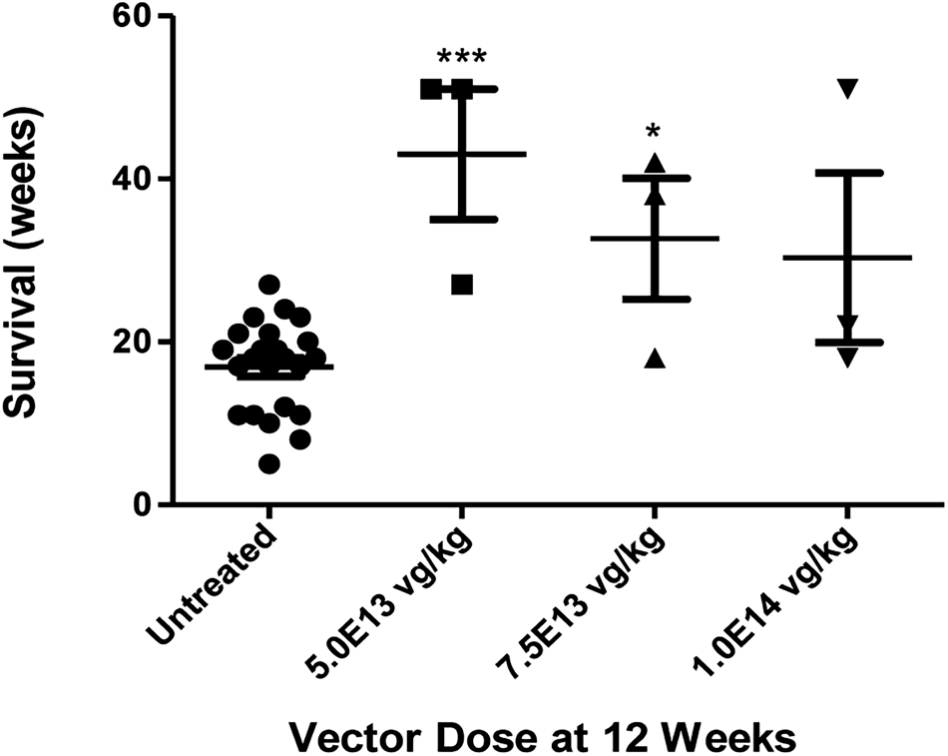
Effect of vector dose on survival of symptomatic AMD cats. AMD cats were treated at 12 weeks of age with a single intracarotid injection of AAVhu.32-expressing fMANB. Treated cats lived longer than untreated cats regardless of vector dose; however, increasing vector dose did not increase survival. Error bars represent mean + SEM. *p < 0.05, ***p < 0.001.

Animals were humanely euthanized once they reached end stage disease. For untreated AMD, the average lifespan is 17 weeks. All three doses increased lifespan relative to untreated AMD cats, however the group treated with the highest dose did not reach significance (Fig. 2). Treatment at 6 weeks of age significantly increased lifespan to an average of 61 weeks [18], however treatment at 12 weeks of age with the equivalent dose increased lifespan to a lesser degree (*P* < 0.001, average lifespan 43 weeks). Treatment with a higher dose of vector did not further extend lifespan. These data suggest that while intracarotid injection of AAVhu.32-fMANB improves survival relative to untreated animals regardless of age at injection, earlier treatment leads to greater extension of lifespan.

Neurological examinations suggest that a less than sufficient amount of therapeutic MANB enzyme is being expressed in the brains of treated cats. To measure the amount of MANB activity in the brain, coronal sections at multiple points along the rostral-caudal axis were measured post-mortem. For all treatment groups, MANB activities above untreated AMD brain sections were detected (Table 1), with the greatest levels of MANB activity measured in the group treated with the 7.5 × 10^13^ vg/kg dose. To determine whether the low MANB activity is due to low transduction of the brain, the number of vector genome copies from coronal sections collected at different intervals along the rostral-caudal axis were also measured (Table 2). The number of vector genomes were generally low; however, a significantly higher number of vector genomes was measured throughout the brain in animals treated with 7.5 × 10^13^ vg/kg vector. In particular, the group treated with 5.0 × 1013 vg/kg vector at 12 weeks had lower MANB activity and MANB vector genome copies throughout the brain than what was measured in AMD cats treated with the same dose at 6 weeks [18].

**Table 1:** MANB activity measured in multiple brain hemisections along the rostral-to-caudal axis.

Treatment Group	Animal	Hemisection					
		1	2	3	4	5	6
Untreated	1	1.065	0.591	0.630	1.210	0.806	0.739
	2	0.519	0.372	0.437	0.585	0.793	0.800
	3	0.631	0.704	0.779	0.957	0.958	0.745
	4	0.528	0.626	0.743	0.684	1.111	0.901
	**Average (+SEM)**	**0.686 (0.129)**	**0.573** **(0.071)**	**0.647** **(0.077)**	**0.859 (0.141)**	**0.917** **(0.075)**	**0.796 (0.037)**
5.0 x 10^13^ vg/kg	1	2.004	2.017	1.717	1.967	2.469	1.604
	2	3.165	2.847	3.073	2.390	2.917	3.116
	3	0.756	0.771	0.749	0.742	0.424	0.576
	**Average (+SEM)**	**1.975** **(0.695)**	**1.878** **(0.603)**	**1.846** **(0.674)**	**1.700** **(0.494)**	**1.936** **(0.767)**	**1.765** **(0.738)**
7.5 ×10^13^ vg/kg	1	3.134	3.437	2.456	2.790	2.797	1.930
	2	4.995	5.368	5.808	7.210	6.592	3.846
	3	3.467	4.027	3.449	4.750	1.711	1.861
	**Average (+SEM)**	3.865^**^ **(0.573)**	4.278^***^ **(0.571)**	3.904^**^ **(0.994)**	4.917^***,++^ **(1.279)**	3.700^*^ **(1.480)**	**2.546** **(0.650)**
1.0 x 10^14^ vg/kg	1	3.157	4.294	2.979	3.935	2.818	1.688
	2	2.229	2.377	2.269	2.691	2.688	1.515
	3	1.972	2.944	2.811	3.544	2.922	2.379
	**Average (+SEM)**	**2.453** **(0.360)**	3.205^*^ **(0.568)**	**2.687** **(0.214)**	**3.390** **(0.367)**	**2.809** **(0.068)**	**1.860** **(0.264)**

Brain fMANB activity in both treated and untreated AMD cats was measured in coronal sections taken at regular intervals along the rostral-caudal axis. Activity is calculated and reported as a percentage of normal.

*p < 0.05; **p < 0.01; ***p < 0.001 compared to untreated animals.

+ +*p* < 0.01 compared to 5.0 ×10^13^ vg/kg group.

**Table 2:** MANB vector genomes measured in multiple brain hemisections along the rostral-to-caudal axis.

Treatment Group	Animal	Hemisection					
		1	2	3	4	5	6
5.0 × 10^13^ vg/kg	1	0.069	0.077	0.232	0.322	0.035	0.143
	2	0.230	0.128	0.072	0.140	0.096	0.052
	3	0.013	0.003	0.007	0.004	0.007	0.007
	**Average (+SEM)**	0.104^*^ **(0.065)**	0.069^***^ **(0.036)**	0.104^**^ **(0.067)**	0.155^**^ **(0.092)**	**0.046** **(0.026)**	**0.067** **(0.040)**
7.5 × 10^13^ vg/kg	1	0.786	0.608	0.669	0.563	0.589	0.291
	2	0.558	0.638	0.706	0.984	1.151	0.792
	3	1.174	0.860	0.808	0.901	0.347	0.372
	**Average (+SEM)**	**0.839** **(0.180)**	**0.702** **(0.079)**	**0.728** **(0.041)**	**0.816** **(0.129)**	**0.696** **(0.238)**	**0.485** **(0.155)**
1.0 × 10^14^ vg/kg	1	0.292	0.247	0.208	0.290	0.254	0.123
	2	0.052	0.144	0.117	0.176	0.129	0.111
	3	0.397	0.293	0.414	0.404	0.345	0.174
	**Average (+SEM)**	0.247^*^ **(0.102)**	0.228^**^ **(0.044)**	0.246^**^ **(0.088)**	0.290^*^ **(0.066)**	**0.243** **(0.063)**	**0.136** **(0.019)**

Brain fMANB vector genome copies in treated AMD cats were measured in coronal sections taken at regular intervals along the rostral-caudal axis. Data is calculated and reported as copies per diploid genome.

*p < 0.05; **p < 0.01; ***p < 0.001 compared to 7.5 × 10^13^ vg/kg group.

Our lab has previously demonstrated that non-invasive imaging can be used to monitor effects of treatment in the living AMD cat brain. Abnormally accumulated mannose-rich oligosaccharides can be monitored in gray matter using MRS [18, 37], and in white matter using DTI [36, 38]. Brains treated at 6 weeks with 5.0 × 1013 vg/kg AAVhu.32-fMANB appeared to be normalized, as evidenced by a significant reduction of the major mannose-containing oligosaccharide peak in MRS and a correction of the leukodystrophy in the DTI [18]. AMD brains treated at 12 weeks of age had partial correction, as evidenced by partial reduction of the major mannose-containing oligosaccharide peak and minimal changes to leukodystrophy (Table 3). Increasing vector dose did not improve the amount of correction.

**Table 3:** MR Spectroscopy and DTI in AMD Cats Treated at 12 Weeks with Intracarotid AAV.hu32-fMANB.

Geno-type	Vector	Titer	*n*	MRI Time Points	MRS Ms/H_2_O ratio (x10^-3^)		DTI Fractional Anisotropy (FA)		
					cerebral cortex	thalamus	external capsule	internal capsule	corpus callosum
Normal	none	NA	3	3	0.14 (0.06)	0.13 (0.05)	0.66 (0.05)	0.68 (0.05)	0.29 (0.04)
AMD	none	NA	5	5	1.96 (0.30)	2.11 (0.31)	0.44 (0.02)	0.46 (0.03)	0.22 (0.02)
AMD Treated	AAV.hu32-fMANB	5 x 10e13	4	10	1.50 (0.41) & *P* = 0.12 # P < 0.01	1.78 (0.13) & *P* = 0.07 # *P* < 0.001	0.47 (0.03) & *P *= 0.12 # *P* < 0.01	0.45 (0.04) & *P* = 0.76 # *P* < 0.001	0.23 (0.02) & *P* = 0.11 # *P* = 0.02
AMD Treated	AAV.hu32-fMANB	7.5 x 10e13	3	5	1.65 (0.16) & *P* = 0.11 # *P* < 0.01	1.82 (0.15) & *P* = 0.12 # *P* < 0.01	0.46 (0.02) & *P* = 0.13 # *P* < 0.01	0.47 (0.03) & *P* = 0.58 # *P *< 0.01	0.24 (0.02) & *P *= 0.11 # *P* = 0.03
AMD Treated	AAV.hu32-fMANB	1.0 x 10e14	3	5	1.70 (0.21) & *P* = 0.21 # *P* < 0.01	1.90 (0.40) & *P* = 0.19 # *P* < 0.001	0.45 (0.05) & *P* = 0.66 # *P* < 0.01	0.46 (0.03) & *P* = 0.83 # *P* < 0.01	0.21 (0.02) & *P *= 0.86 # *P* = 0.01

MRS is expressed as ratios of areas under the curve (integration) of metabolites to water for the oligosaccharide peaks (± SD). The DTI measure used is fractional anisotropy (±SD).

& *T* test between untreated and treated groups.

# *T* test between normal controls and treated groups.

**Fig. 3:**
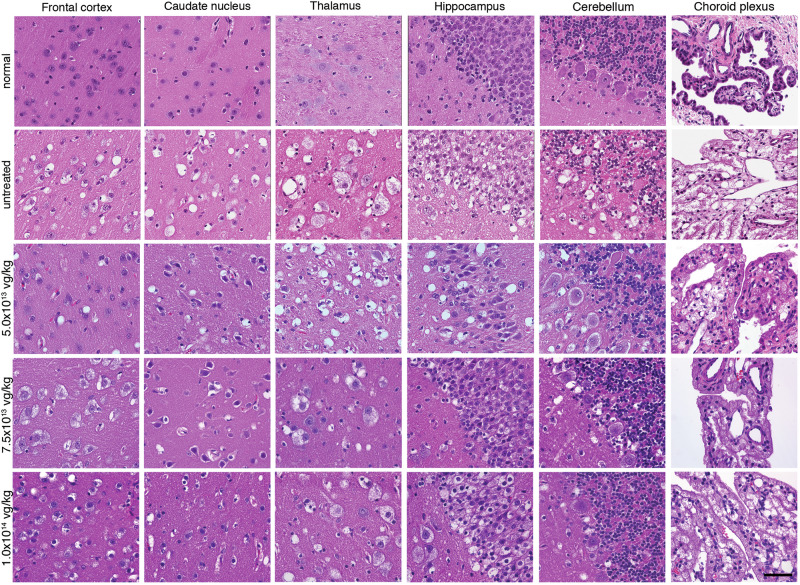
Effect of vector dose on clearance of lysosomal storage lesions in the brain of symptomatic AMD cats. Representative hematoxylin and eosin-stained brain sections of normal, untreated, and AAVhu.32-fMANB-treated cats with increasing doses in major brain structures. Partial storage reduction is seen in all treatment groups, but there was little difference between the treatment groups. Scale bar, 50 μm.

**Fig. 4:**
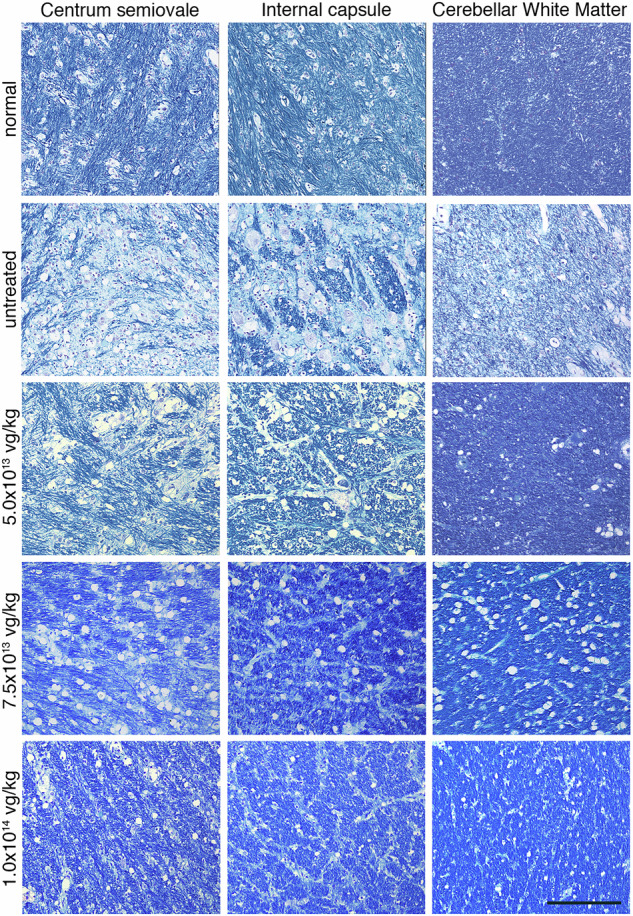
Effect of vector dose on major white matter structures of symptomatic AMD cats. Representative Luxol fast blue stained brain sections of normal, untreated, and AAVhu.32-fMANB-treated cats at increasing vector doses. Partial improvement of myelin intensity is seen in all treatment groups, however, myelin intensity does not improve by increasing vector dose. Scale bar, 100 μm.

The MRS and DTI data is consistent with histological staining of brain sections. Whereas animals treated at 6 weeks achieved near complete correction of storage lesions throughout the brain as visualized by hematoxylin and eosin staining [18], sections from animals treated at 12 weeks show only partial correction of oligosaccharide storage throughout the brain relative to untreated cats. Furthermore, there was no apparent difference between the three treatment groups (Fig. 3). Luxol fast blue stained sections also show some improvement in treated cats compared to untreated animals, but the extent of myelin distension and splitting is comparable between treatment groups (Fig. 4). All groups show less improvement than what was observed in animals treated at 6 weeks [18].

As the genetic defect characteristic of AMD is found in all tissues, storage can be found in tissues outside the CNS. MANB activity was measured in liver, kidney, heart and spleen samples following intracarotid infusion of vector. MANB activity in the liver was the only tissue that was elevated above untreated, and this increase was only significant in the group treated with 5.0 × 10^13^ vg/kg vector (Table 4). Furthermore, the MANB activity levels in the 5.0 x 10^13^ vg/kg group were comparable to that measured in tissues in animals treated at 6 weeks of age [18]. MANB activity levels in other organs tested were similar to untreated animals. Vector genome copies were elevated but variable in liver samples (Table 5), and low levels of vector genomes were found in other tissues.

**Table 4:** MANB activity measured in tissue samples from select organs.

Treatment Group	Animal	Organ			
		Liver	Kidney	Heart	Spleen
Untreated	1	0.095	0.026	1.740	0.171
	2	0.509	0.136	3.235	1.310
	3	0.647	0.084	0.868	1.085
	4	0.147	0.046	1.729	0.966
	5	0.251	0.099	0.692	0.658
	6	0.131	0.079		0.389
	**Average (+SEM)**	**0.297** **(0.275)**	**0.078** **(0.016)**	**1.653** **(0.450)**	**0.763** **(0.178)**
5.0 × 10^13^ vg/kg	1	3.707	0.224	3.127	0.271
	2	6.373	0.257	5.842	
	3	1.066	0.137	0.869	
	**Average (+SEM)**	3.716^**^ **(1.532)**	**0.206** **(0.036)**	**3.279** **(1.438)**	**0.271**
7.5 × 10^13^ vg/kg	1	1.517	0.176	1.554	0.272
	2	0.677	0.109	2.482	0.137
	3	0.768	0.690	2.470	0.066
	**Average (+SEM)**	**0.987** **(0.266)**	**0.325** **(0.183)**	**2.169** **(0.307)**	**0.158** **(0.061)**
1.0 × 10^14^ vg/kg	1	1.319	0.271	2.418	1.015
	2	0.640	0.081	1.277	0.215
	3	0.864	0.071	1.243	0.691
	**Average (+SEM)**	**0.941** **(0.200)**	**0.141** **(0.065)**	**1.646** **(0.386)**	**0.640** **(0.232)**

Brain fMANB activity in both treated and untreated AMD cats was measured in samples collected from a selection of non-CNS tissues. Activity is calculated and reported as a percentage of normal.

**p < 0.01 compared to untreated animals.

**Table 5:** MANB vector genomes measured in select tissues.

Treatment Group	Animal	Organ			
		Liver	Kidney	Heart	Spleen
5.0 ×10^13^ vg/kg	1	70.910	1.581	8.127	0.023
	2	65.090	2.289	6.623	
	3	11.097	0.023	0.226	
	**Average (+SEM)**	**49.032** **(19.042)**	**1.298** **(0.669)**	**4.992** **(2.422)**	**0.023**
7.5 ×10^13^ vg/kg	1	2.369	0.200	2.531	0.074
	2	28.443	2.649	13.492	0.667
	3	80.637	0.831	3.764	0.332
	**Average (+SEM)**	**37.150** **(23.010)**	**1.227** **(0.734)**	**6.596** **(3.466)**	**0.358** **(0.172)**
1.0 ×10^14^ vg/kg	1	4.400	0.226	0.554	0.180
	2	67.994	0.406	3.318	0.683
	3	53.597	0.853	8.962	0.998
	**Average (+SEM)**	**41.997** **(19.252)**	**0.495** **(0.187)**	**4.278** **(2.474)**	**0.621** **(0.238)**

Brain fMANB vector genome copies in treated AMD cats were measured in tissue samples taken from a selection of non-CNS organs. Data is calculated and reported as copies per diploid genome.

No comparisons are significant.

Due to the high vector doses used in the study, vector-induced toxicity is a concern. To assess whether liver or kidney toxicity was present following systemic vector injection, clinical chemistry assays were performed on serum samples taken post-injection. Serum chemistry values from AMD cats were within the control range of 26 age-matched normal cats from our colony, with a small elevation of ALT and AST in cat 3 of the 7.5 × 1013 vg/kg group, suggesting that AAVhu.32-fMANB injection did not result in significant peripheral organ toxicity (Table S1). Despite the presence of some MANB activity in the liver, hematoxylin and eosin-stained liver and kidney sections were histologically similar to those from untreated animals (Fig. S1). However, there were no signs of vector-induced toxicity histologically.

## Discussion

Multiple studies have highlighted the benefits of early therapeutic intervention in the treatment of lysosomal storage diseases. However, diagnosis and treatment in humans is often delayed until the patient is symptomatic with advanced pathology. We have previously demonstrated in α-mannosidosis (AMD) cats that response to AAV1-fMANB delivered to the cisterna magna (CM) decreases at later stages of disease progression [29]. In the current study, we performed a dose escalation study in AMD cats with advanced pathology to determine if there is any therapeutic benefit to increased vector dose. Vector was administered via the carotid artery, which allows for higher volumes (and therefore higher vector doses) to be administered than what was possible in the CM study.

We started with a vector dose that previously generated complete correction throughout the brain in AMD cats injected at 6 weeks [18]. A 12-week timepoint was chosen to simulate treatment at an advanced diseased state. When compared to animals treated at 6 weeks, the animals treated with 5.0 × 10^13^ vg/kg AAVhu.32-fMANB at 12 weeks had a worse prognosis, and increasing vector dose did not provide substantial improvements to clinical outcomes. While 5.0 × 10^13^ vg/kg AAVhu.32 was sufficient for complete correction of the AMD cat brain when administered at 6 weeks, the same vector dose at 12 weeks produced partial correction as measured by MRS and visualized by histology. Increasing vector dose did not noticeably improve correction throughout the brain. Consistent with these findings, MANB activity in the brains of cats treated at 12 weeks was much lower than that measured in animals treated at 6 weeks with the same dose [18]. Increasing vector dose produced a modest increase in brain MANB activity, however MANB activity levels remained lower than that measured in AMD cats treated at 6 weeks. The lower MANB levels in the CSF and brains of cats treated at 12 weeks may be the result of less efficient crossing of the BBB due to the advanced disease stage. The mechanism by which AAVhu.32 crosses the BBB is not currently known, but it is possible that as storage accumulates in cells this mechanism becomes disrupted or less efficient.

A decreased response to treatment in the presence of advanced disease is supported by studies in several other disease models. Studies in mouse models of X-linked juvenile retinoschisis and X-linked retinitis pigmentosa demonstrated a reduced effect when initiating it in older animals, however in the latter study, the treated group performed better than animals receiving vehicle alone [39, 40]. It is also possible that therapy becomes more inconsistent in a more advanced disease state. A study in aged Parkinsonian rats demonstrated a stratification of treated animals into “responder” and “non-responder” groups following treatment with AAV-Ca_V_1.3-shRNA vector [41]. In that study, a uniform response to vector was reported in young male rats, however, the response was more variable in aged rats.

There are a few studies which suggest that treatment of advanced disease may be possible. A study in a mouse model of Wilson disease, showed a decrease in therapeutic efficacy following treatment of animals with more advanced disease [42]. However, an optimized “mini” version of the protein was capable of restoring copper homeostasis even in animals with advanced disease. In MPS I dogs, injection of AAV-IDUA into the cornea produced resolution of corneal clouding even in animals with advanced disease [43]. While these studies suggest that in some cases treatment of advanced disease may be possible, this outcome may be reliant on the nature of the disease being treated.

In the AMD cat, the optimal window for treatment is when symptoms first become apparent, as efficacy declines as the disease advances [29]. Here, we provide evidence that the decrease in efficacy cannot be overcome simply by increasing vector dose. The lack of a dose-response relationship is likely a function of the advanced stage of disease and progression of the pathology in the cells the vector interacts with to cross the BBB. Intracisternal treatment also decreased in efficacy when treated at later stages of disease [29]. Interestingly, reduced vector delivery to the CNS did not result in a corresponding increase in transduction outside the CNS. The lack of a dose-response relationship suggests that the pathology present at late-stage disease is less reversible. Nevertheless, partial correction extended lifespan compared to untreated AMD cats. This suggests that even late-stage initiation of treatment may be clinically beneficial to patients by slowing or stabilizing the progressive degenerative course of the disease. Collectively, the data presented here and in our previous work further highlight the importance of early diagnosis and treatment of lysosomal storage diseases such as α-mannosidosis.

## Supplementary information

Supplemental text

## Data Availability

All data and supporting materials are available within the article and supplemental information. Raw data can be provided by the corresponding author upon reasonable request.
