# Supplementary material for: Clinical response to systemic AAV gene therapy in a large animal model of late-stage lysosomal storage disease
Source: Gene Ther. 2026 May 4;33(4):495–503. doi: 10.1038/s41434-026-00618-0 (PMC13472877; doi:10.1038/s41434-026-00618-0)
Supplement: Supplementary file 1 [file 41434_2026_618_MOESM1_ESM.docx]

|  | | | **Dose Group** | | | | | | | | |
| --- | --- | --- | --- | --- | --- | --- | --- | --- | --- | --- | --- |
| **Test** | **Normal cats in colony (n=26)** | | **5.0 x 10^13^ vg/kg** | | | **7.5 x 10^13^ vg/kg** | | | **1.0 x 10^14^ vg/kg** | | |
| **Liver Function** | **Range** | **Mean** | 1 | 2 | 3 | 1 | *2* | 3 | 1 | *2* | 3 |
| ALT | 27-99 | 52.4 | 27 | 29 | ND | 75 | 52 | 118 | 67 | 67 | 76 |
| AST | 11-84 | 22 | 22 | 30 | ND | 40 | 40 | 94 | 44 | 42 | 48 |
| Alk. Phos. | 23-197 | 93.9 | 24 | 22 | ND | 55 | 50 | 104 | 28 | 77 | 72 |
| **Kidney Function** |  | | | | | | | | | | |
| BUN | 12-35 | 23.7 | 30 | 33 | ND | 30 | 32 | 28 | 32 | 31 | 34 |
| Creatinine | 0.4-1.4 | 0.9 | 1.2 | 0.9 | ND | 1.0 | 0.6 | 1.0 | 1.3 | 0.8 | 1.2 |
|  | | | | | | | | | | | |
| **Age** |  |  | 51 | 51 | ND | 42 | 18 | 38 | 51 | 22 | 18 |

**Table S1 Serum chemistry of cats used in study**

Blood was collected from treated cats when the animal reached the humane endpoint and analyzed for several markers of liver and kidney function. Columns contain values for each animal in a treatment group. ND - no bloodwork done one individual in 5.0 x 10^13^ vg/kg dose group due to COVID-19 pandemic.

**Figure S1. Treatment does not correct storage in liver or kidney of treated cats.** Representative hematoxylin and eosin-stained liver sections of normal, untreated, and AAVhu.32-fMANB-treated cats. Little to no improvement of storage is seen in treated cats when compared to untreated AMD cats. Scale bar = 50 μm.
